# Oral heparin: status review

**DOI:** 10.1186/1477-9560-4-6

**Published:** 2006-05-10

**Authors:** Ehud Arbit, Michael Goldberg, Isabel Gomez-Orellana, Shingai Majuru

**Affiliations:** 1 Emisphere Technologies Inc. 765 Old Saw Mill River Road. Tarrytown, NY 10591, USA

## Abstract

Unfractionated heparin and low molecular weight heparin are the most commonly used antithrombotic and thromboprophylactic agents in hospital practice. Extended out-of-hospital treatment is inconvenient in that these agents must be administered parenterally. Current research is directed at development of a safe and effective oral antithrombotic agent as an alternative for the effective, yet difficult to use vitamin K antagonists. A novel drug delivery technology that facilitates transport of drugs across the gastrointestinal epithelium has been harnessed to develop an oral dosage form of unfractionated heparin. Combining unfractionated heparin with the carrier molecule, sodium N-(8 [2-hydroxybenzoyl]amino) caprylate, or SNAC has markedly increased the gastrointestinal absorption of this drug. Preclinical and clinical studies to-date suggests that oral heparin-SNAC can confer a clinical efficacious effect; further confirmation is sought in planned clinical trials.

## Introduction

The current use of anticoagulants is extensive and it is estimated that 0.7% of the population in the Western world receive these drugs [1]. Broader indications for anticoagulants and their increased use in the outpatient settings as well as for long-term dosing has stimulated renewed interest in developing oral anticoagulant and antithrombotic agents. At present, the cornerstone of oral anticoagulants are the vitamin K antagonists, of which the coumarin derivative warfarin, (Coumadin) is the most widely used. It has been used clinically for more than 50 years, and has consistently demonstrated that adequate dosing virtually eliminates recurrent venous thrombosis [2,3]. Nevertheless, warfarin has serious drawbacks that require steady vigilance on the part of clinicians. These drawbacks include significant drug-drug and food-drug interactions, a slow onset and offset of effect, and a narrow therapeutic index. Because of the inherent variability in response over time and the consequently unpredictable pharmacodynamics of the drug, frequent monitoring is necessary, an inconvenience for the large number of patients who take it chronically. Even with optimal warfarin monitoring in patients with atrial fibrillation (AF), therapeutic anticoagulation is achieved only half the time [4,5]. Because of this, it is estimated that at least half the patients with nonvalvular AF who are eligible for warfarin therapy do not receive it [6,7]. A forthcoming oral direct thrombin inhibitor, ximalegatran, was anticipated as a replacement for warfarin, and study results were promising [8]. However, concerns with regard to hepatotoxicity with long term use have been raised [9].

A medical need still remains for a safe and effective oral anticoagulant that is easier than warfarin for physicians and patients to use on a long-term basis. In response to this unmet need a novel oral drug delivery technology that enables poorly absorbed molecules to be absorbed through the gastrointestinal tract was harnessed to devise an oral form of unfractionated heparin (UFH) [10,11]. Theoretically an oral form of heparin or low molecular weight heparin (LMWH) administered at a fixed dose, twice or thrice daily, free of the need for frequent coagulation monitoring or dose adjustments, and with a low potential of drug-drug and food-drug interactions would embody the desirable anticoagulant profile for long- term oral use.

Heparin was discovered more than 80 years ago by a medical student, Jay McLean who found that an extract of dog liver prolonged the time required for plasma to clot *ex vivo *[12]. It has been in clinical use for over 50 years and has withstood the test of time in terms of both efficacy and safety. Heparin remains one of the most important anticoagulant drugs in current clinical use and is the drug of choice when rapid effect is desired such as in the intensive care setting, during surgery and for patients with renal failure. Over the past few decade LMWH preparations, which are fragments of UFH produced by controlled enzymatic or chemical depolymerization have risen in popularity. LMWHs have a more predictable pharmacokinetic profile than UFH can be administered by subcutaneous injection (s.c.) once or twice daily and do not require laboratory monitoring. This simplified regimen with LMWHs has widened the range of their clinical applications and paved the way for LMWHs to supersede UFH for most indications that necessitate out-patient and long-term treatment. A major disadvantage of both UFH and LMWH therapy lies in the fact that the size and charge of these molecules make (.)parenteral administration a necessity.

The combination of UFH with a delivery agent, the basis of the newly advanced drug delivery technology employed, achieves heparin absorption when administered orally, in amounts adequate for therapeutic purpose and is currently in clinical trials [13-15]. Presented is a status review of oral UFH including a brief description of the technology employed and the results of clinical studies thus far conducted with oral UFH.

### The technology

**Figure 1:**
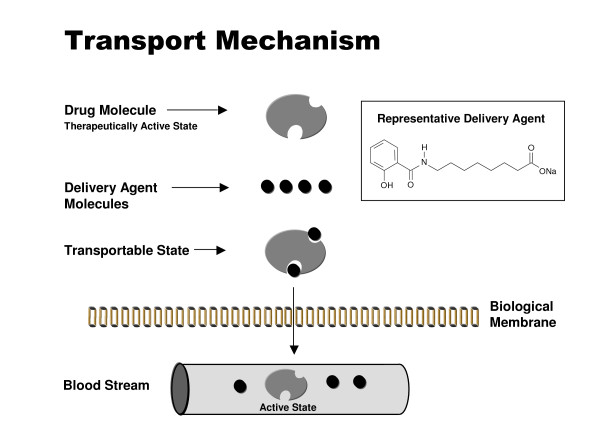
**Schematic depiction of drug delivery technology**. Carrier molecule (delivery agent) associates with drug molecule to create a transportable complex (lipophilic). Because of the weak association, carrier and drug dissociate by simple dilution on entering the blood circulation.

For a drug to be absorbed from the gastrointestinal tract and retain its efficacy, it must withstand the harsh chemical and biological milieu within the gastrointestinal tract. In addition, to be absorbed it must have certain specific physicochemical properties. Among these are a suitable molecular weight (typically below 500–1000 daltons), pKa (a measure of the degree of acidity or basisity), degree of lipophilicity (log D) as well as proper solubility. Most drugs are either weak acids or weak bases, and under normal conditions only the nonionized fraction (the most lipophilic) crosses biological membranes, except where active transport is involved. The new technology advanced to overcome these limitations is based on carrier molecules, which are comprised of small organic compounds (200–400 Da). These carriers interact with the drug molecules to create a weak, non-covalent association, the drug remaining chemically unmodified [16-18]. The carriers possess hydrophobic moieties that on association with the drug molecules create a more lipophilic drug/carrier complex, enabling transport across the epithelial membrane [19-21]. Because of the weak association between carrier and drug, the interaction is reversible, and occurs spontaneously by simple dilution on entering the blood circulation (Figure 1). Studies have shown that the carriers enable the systemic absorption of the drug via transcellular absorption, a common drug absorption pathway, without compromising the integrity of the intestinal epithelium [17,22-24].

The carrier selected to develop oral forms of heparin is sodium *N*-(8 [2-hydroxybenzoyl]amino) caprylate, or SNAC [10,11]. Heparin plasma levels attained with oral heparin-SNAC (aPTT, anti-factor Xa assay) reached the therapeutic range in humans, and were found to be effective in reducing the incidence of DVT in animal models of venous thrombosis [13,14,25,26].

### Heparin and its indications

In preclinical developmental studies, the carrier-based technology was able to deliver orally both UFH and LMWH [27-29]. A number of factors were considered in the deliberation to advance UFH as the first oral heparin candidate, not the least of which was the fact that the LMWHs are currently still proprietary products.

Heparin, a naturally occurring glycosaminoglycan, in addition to its anticoagulant effect is also implicated in an ever growing number of physiological and pathological processes such as inflammation, immune cell migration, tumor cell metastasis, and smooth muscle cell (SMC) proliferation among others [30-36]

The drug has a relatively rapid clearance, ranging from 30 minutes to 2.5 hours, depending on the dose, which allows for relatively easy and frequent adjustments of therapeutic level. Heparin dose can be readily measured and monitored by a point-of-care assay using the activated partial thromboplastin time (aPTT) [37,38]. On starting heparin there is no initial hypercoagulable state or transition phase, and the anticoagulant effect can be reversed with protamine if needed. Heparin is cleared and degraded primarily by the reticuloendothelial system and therefore is the drug of choice when full therapeutic anticoagulation therapy is required in patients with severe renal failure [39].

An important activity of heparin and LMWHs is the release of tissue factor pathway inhibitor (TFPI) from endothelial cells [40]. Release of TFPI appears to be related to the heparin chain length (MW) and degree of sulfation, which are highest in UFH. As the chain length shortens, the release of TFPI decreases [41-43]. Over-expression of tissue factor, in addition to its procoagulant effect, is also implicated in the pathophysiology of sepsis, acute lung injury, disseminated intravascular clotting angiogenesis and cancer [44-47]. Incidentally, the long half-life of TFPI is also believed to account for the prolonged protection from thrombosis conferred by heparins, rather than by the mere activation of anti-factor Xa and anti-factor IIa, which have a relatively short plasma half-life.

LMWHs have largely supplanted UFH in the clinic because of their more predictable pharmacokinetics and essentially a safety profile comparable to that of UFH [48-58]. After subcutaneous injection, LMWHs achieve a higher bioavailability and a longer half-life (roughly 4 hours), allowing for a sustained effect with once or twice daily fixed or weight-adjusted dosages and monitoring of anticoagulant levels is usually not required.

It is common to assess clinical efficacy of novel anticoagulants in DVT prophylaxis studies in high risk surgical patients such as those after total hip replacement surgery. The reason being that the prevalence of the DVT in these patients is high and the duration of prophylaxis (postoperative treatment) is relatively short. Nevertheless, the intended clinical indications for anticoagulant drugs, with a few examples of which will be enumerated below, are generally aimed at a broader patient population.

Oral heparin will be a particularly appropriate anticoagulant for indications that require prophylaxis or treatment for extended periods, months or indefinitely [59]. Extended prophylaxis is indicated as an example in situations such as when thrombosis is idiopathic or associated with a continuing risk factor which cannot be identified and eliminated, [60,61]. Indefinite anticoagulant therapy is generally indicated in AF patients and in patients with prosthetic heart valves, in patients with idiopathic recurrent proximal vein thrombosis, thrombosis complicating malignancy, and in patients with homozygous factor V Leidengenotype, the antiphospholipid antibody syndrome, or deficiencies of antithrombin III, protein C, or protein S [62-64].

Anticoagulation in women of gestational age with prospects of pregnancy requiring anticoagulation pose an especially complex situation where oral heparin may have an important role. Anticoagulant therapy during pregnancy is indicated for prevention and treatment of DVT, for the prevention of and treatment of systemic embolism in patients with mechanical heart valves and for the prevention of pregnancy complications in women with antiphospholipid antibodies or other thrombophilia and previous pregnancy complications [65,66]. Coumarin derivatives cross the placenta and can produce a characteristic embryopathy with first-trimester exposure and, less commonly, central nervous system abnormalities and fetal bleeding with exposure after the first trimester [67]. For this reason, it has been recommended that warfarin therapy be avoided during the first trimester of pregnancy and, except in special circumstances, avoided entirely throughout pregnancy. Neither UFH nor LMWH cross the placenta and therefore these agents are safe in pregnancy.

### Clinical and formulation development of oral heparin

The objectives of the initial clinical studies with oral heparin-SNAC were to demonstrate a) the safety and tolerability of unformulated solutions of heparin/SNAC combination b) that heparin without SNAC administered orally is not absorbed, c) that SNAC by itself has no effects on coagulation and d) to establish the minimal dose of oral heparin required to have a pharmacological effect when combined with maximal safe dose of SNAC. A single dose of 30,000 USP Heparin Units or SNAC alone at doses ranging from 1.4 to 10.5 g were administered orally. Measurements of anticoagulation (aPTT, TFPI and anti-Factors IIa and Xa levels) did not change following administration of either Heparin alone or SNAC alone. In a subsequent study, an unformulated solution of SNAC (10.5 g) in combination with 10,000, 20,000 or 30,000 USP heparin units were administered orally. Elevated aPTT, anti-Factors IIa and Xa levels were observed following oral administration of 20,000 Units and 30,000 Units in combination with 10.5 g of SNAC, demonstrating that SNAC facilitates the absorption of heparin when administered as heparin/SNAC solution in human subjects [13].

**Figure 2:**
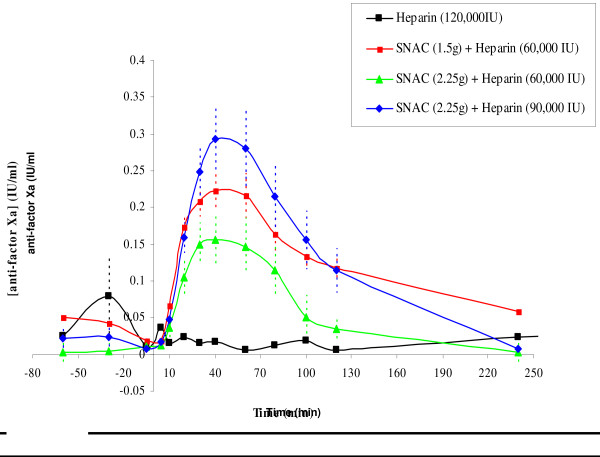
**Anti-Factor Xa levels**. Blood levels of Anti-Factor Xa after oral administration of heparin/SNAC in solution to healthy male volunteers (mean ± SEM). A dose response was observed. Heparin alone was not absorbed.

A taste-masked SNAC/Heparin oral solution formulation was developed and evaluated at a fixed dose (2.25 g) of SNAC in combination with escalating doses (30,000, 60,000, 90,000 and 150,000 Units) of heparin. Following oral administration the subjects exhibited dose-dependent prolongations in mean aPTT and anti-Factor Xa as shown in Figure 2. Safety, tolerability and pharmacokinetics of the taste-masked formulation were further evaluated in a study comprising 12 healthy adults (age 18–59) and 12 healthy elderly (≥ 60 years old) volunteers. An additional objective was to compare the disposition (*E*_max_, AUEC_0–8_) of oral heparin/SNAC relative to s.c. injection of 5000 Units heparin (Heparin Sodium Injection, USP; Wyeth-Ayerst, Philadelphia, PA, USA). In this open-label study, subjects received 15 mL of formulated, taste-masked, oral heparin/SNAC solution (90 000 Units/2.25 g) every 8 hr, either 1 hour before or 2 hours after meals, for a total of 16 doses over 6 days. The study showed that a larger increase in aPTT occurred after oral heparin/SNAC than s.c. UFH. A trend toward greater maximal increase above baseline (*E*_max_) and area under the effect curve (AUEC) for aPTT and anti-FXa was observed in elderly compared to younger subjects which was consistent with a lower (9 kg) mean body weight. The half disappearance time (*t *_1/2_) was the same in adult and elderly subjects when measured by aPTT, but there was a trend towards prolonged anti-FXa effect in elderly subjects (0.5 vs. 0.38 hours on day 1 and 0.62 vs. 0.38 hours on day 6). There was no significant difference in *t*_1/2 _between the last and first doses.

In a phase II clinical trial 123 patients undergoing total hip replacement were randomized to receive either oral heparin/SNAC (1.5 g SNAC/60.000 units UFH or 2.25 g SNAC/90.000 units UFH) as a taste masked oral solution or 5000 Units UFH s.c every 8 hours. Patients received study medication for 5 days and were observed for 35 days following surgery. Efficacy assessment was based on day 5 bilateral full leg venous ultrasound and patients were followed for symptomatic VTE events for 35 days. The study revealed that major bleeding events (overall 3.3%) and VTE (overall 4.9%) were not different in either of the two dosages of oral heparin/SNAC or the s.c. heparin group. This study provided the thrust for applying the tested regimens of heparin/SNAC in a Phase III efficacy study.

An international, multi-center phase III thromboprophylaxis trial in 2264 patients with the objective to compare safety and efficacy of two oral doses of UFH to a standard subcutaneous LMWH regimen (PROTECT trial) in patients undergoing elective hip surgery was conducted. The clinical trial was randomized, double-blind (double-dummy: placebo oral or injection). Oral heparin prophylaxis was initiated 4–6 hours postoperatively and continued through the whole evaluation period (27–30 days), while enoxaparin was initiated 12–24 hours postoperatively and was administered for 10 days followed by placebo until the final evaluation. Oral heparin/SNAC solution, low dose 60,000 IU/1.5 g SNAC (ldSNAC) and high dose 90,000 IU/2.25 g SNAC (hdSNAC) were administered trice daily. Subcutaneous LMWH (enoxaparin, Aventis Pharam, Bridgewater, NJ, USA) 30 mg twice daily for 10 days was started 12–24 hours postoperatively and followed by an identical subcutaneous placebo regiment (double dummy) for up to a total of 27 – 30 days. The primary end point was to demonstrate superiority of oral heparin over s.c enoxaparin in reducing the DVT rate as detected by bilateral ascending contrast venography at day 27–30. For each treatment group 743 patients were required to obtain 90% power for analysis of the deep-vein thrombosis (DVT) rates (type I error of 0.025) assuming an interpretable venogram rate of 65%. Baseline characteristics on entry were comparable among the three groups. The rationale for choosing superiority was based on the fact that a procoagulant state persists for at least 4 weeks after THR and the vast majority of DVTs occur after hospital discharge, between days 7 and 21 [68]. It has been theorized that prolonged prophylaxis with an equivalent efficacious drug to enoxaparin, such as oral heparin, would confer superiority of anticoagulant effect over the 30 day study duration. Interpretable venogram was obtained in 64% of patients and included a total of 752, 767, and 745 patients who were randomized to the ldSNAC, hdSNAC and LMWH/placebo groups respectively. Fewer proximal and total DVT's and pulmonary embolisms were observed in patients treated with enoxaparin (117 of 449, 26.1%) and high dose heparin-SNAC (137 of 461, 29.7%) as compared to low dose heparin-SNAC (155 of 488, 31.8%) treated patients (the absolute differences and 95% CI for ldSNAC and hdSNAC compared with LMWH were: 5.7% [0.1% to 11.7% CI] and 3.7% [-1.9% to 9.7% CI]). Proximal DVT or PE occurred in 90 of 485 patients (18.6%) on ldSNAC, 64 of 465 patients (13.8%) on hdSNAC and 57 of 450 patients (12.7%) treated with s.c. LMWH (p = 0.013 comparing ldSNAC to s.c. LMWH and p = 0.045 comparing hdSNAC to ldSNAC) [69]. In terms of safety, the incidence of major/minor bleeding was 0%/1.9% of 728 low dose heparin/SNAC patients, 0.7 %/2.0% of 736 high-dose SNAC/heparin patients, and 0.4%/1.5% of 716 enoxaparin patients. The incidence of PE was very low and not different for all three treatment groups (0.2% – 0.8%). Wound hematomas occurred in 14 (1.9%) of ldSNAC treated patients, 17 (2.3%) of hdSNAC treated patients, and 21 (2.9%) of the s.c. LMWH group, with "complicated" hematomas in 3 (0.4%), 7 (1.0%), and 6 (0.8%) of patients treated with ldSNAC, hdSNAC and s.c. LMWH, respectively. Two patients who suffered thombocytopenia received prophylaxis with intravenous UFH. This study did not meet its primary end point, likely due to a suboptimal dosage form and a poorly tasting liquid formulation; however, it provided proof of concept that heparin delivered by the oral route had potent antithrombotic activity. The study was the first to document in a large patient population that oral heparin can reduce the frequency of postoperative VTE with low frequency of bleeding complications in patients undergoing total hip replacement surgery [15].

A posthoc analysis was undertaken in all patients (a total of 299) enrolled for the study in the US, Canada and Australia, which were sites where compliance and adherence to study protocol could be confirmed. In this analysis a statistical significant reduction in the incidence of DVT was observed in patients assigned to high dose oral heparin compared to the s.c. LMWH group (Table 1). We recognize the limitations of a posthoc analysis approach; nonetheless, the observation of an improved outcome with oral heparin as compared to parentral s.c. LMWH provides impetus for further studies of this product.

**Table 1:** PROTECT Trial, post-hoc analysis of ITTIV population

ITTIV population N = 299 (includes all patients from USA, Canada/Australia)	Low dose oral-heparin (60,000 IU/1.5 g SNAC) 30 days)	High dose oral-heparin (90,000 IU/2.25 g SNAC) 30 days	Injectable LOVENOX ^® ^30 mg s.c b.i.d.)10-day followed by placebo (to day 30)
Total DVT rate	27%	18%	28%
Relative Risk Reduction	4%	55%	

**Figure 3:**
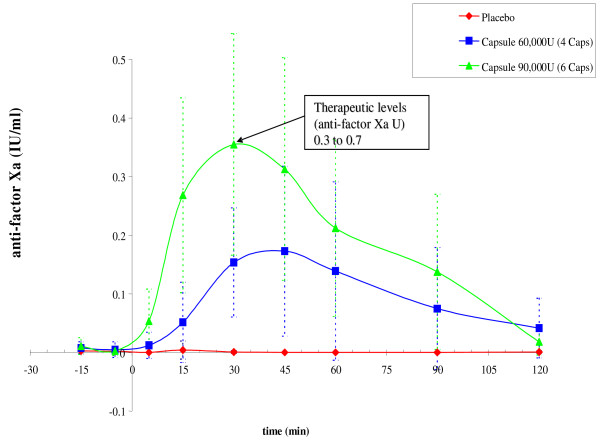
**Anti-Factor Xa levels following oral administration of heparin/SNAC in hard-gelatin capsules**. The study was done on healthy male volunteers (mean ± SD, n = 10, crossover study). Therapeutic levels were attained. Prophylactic levels of anti-factor Xa were only slightly elevated.

The results from the PROTECT trial made clear the need to overcome the adverse taste of the oral heparin formulation and attain a more patient-friendly formulation of heparin/SNAC with the focus being a solid dosage form. Several solid dosage forms were evaluated and a solid oral dosage form that achieved plasma anti-factor Xa levels (Figure 3), comparable to those for the oral solution formulation in healthy volunteers was successfully developed [70]. The clinical development of oral heparin will continue with the evaluation of an optimized solid dosage form and plans are currently underway to evaluate the new heparin/SNAC solid dosage form in a thromboprophylaxis study.

## Conclusion

Although UFH has been in use for decades, attempts to convert either UFH or LMWH to an oral dosage form have been discouraging. Recent studies have shown that the addition of a carrier molecule such as SNAC can facilitate the enteric absorption of both UFH and LMWH and reach levels that are adequate for both prevention and treatment of venous thromboembolic disease in animals and humans. In a phase III randomized clinical trial, it was demonstrated that oral heparin-SNAC reduced the incidence of proximal venous thrombosis in patients undergoing hip replacement surgery in a dose-dependent fashion compared with standard LMWH given subcutaneously. This proof of concept demonstration warrants further studies with oral UFH and LMWH, particularly in a solid dosage form. The oral route still remains the safest, most convenient, most economical and the one which fosters the greatest patient compliance and adherence, virtues that are likely to translate to better health care.

As with all new therapeutic agents there is always concern of new and unexpected toxicity being uncovered over time and with wider use. UFH and LMWH have been in use for decades. Although they are associated with undesirable effects including bleeding, HIT and osteoporosis, these are well known, recognized, managed and it is unlikely that new side effects will be uncovered with further use of these agents.
